# Machine Learning Achieves Pathologist-Level Coeliac Disease Diagnosis

**DOI:** 10.1056/AIoa2400738

**Published:** 2025-03-27

**Authors:** F. Jaeckle, J. Denholm, B. Schreiber, S. C. Evans, M. N. Wicks, J. Y. H. Chan, A. C. Bateman, S. Natu, M. J. Arends, E. Soilleux

**Affiliations:** 1 Department of Pathology, University of Cambridge, Tennis Court Road, CB2 1QP, Cambridge, England, UK; 2 Lyzeum Ltd, Cambridge, CB1 2LA, England, UK; 3 Department of Applied Mathematics and Theoretical Physics, University of Cambridge, Wilberforce Road, CB3 0WA, Cambridge, England, UK; 4 Edinburgh Pathology & Centre for Comparative Pathology, Institute of Genetics & Cancer, University of Edinburgh, Crewe Road, Edinburgh EH4 2XR, UK; 5 Cambridge University Hospitals NHS Foundation Trust, Cambridge, UK; 6 University Hospital Southampton NHS Foundation Trust, Southampton, UK; 7 University Hospital of North Tees, North Tees and Hartlepool NHS Foundation Trust, Hardwick, Stockton on Tees, England, UK

## Abstract

**Background:**

The diagnosis of coeliac disease (CD), an autoimmune disorder with an estimated global prevalence of around 1%, generally relies on the histological examination of duodenal biopsies. However, inter-pathologist agreement for coeliac disease diagnosis is estimated to be no more than 80%. We aim to improve coeliac disease diagnosis by developing a novel, accurate, machine-learning-based diagnostic classifier.

**Methods:**

We present a machine learning model that diagnoses the presence or absence of coeliac disease from a set of duodenal biopsies representative of real-world clinical data. Our model was trained on a diverse dataset of 3,383 -slide images (WSIs) of H&E-stained duodenal biopsies from four hospitals featuring five different WSI scanners along with their clinical diagnoses. We trained our model using the multiple-instance-learning paradigm in a weakly-supervised manner with cross-validation and evaluated it on an independent test set featuring 644 unseen scans from a different regional NHS Trust. Additionally, we compared the model’s predictions to independent diagnoses from four specialist pathologists on a subset of the test data.

**Results:**

Our model diagnosed coeliac disease in an independent test set from a previously unseen source with accuracy, sensitivity, and specificity exceeding 95% and an area under the ROC curve exceeding 99%. These results indicate that the model has the potential to outperform pathologists. In comparing the model’s predictions to diagnoses on unseen test data from four independent pathologists, we found statistically indistinguishable results between pathologist-pathologist and pathologist-model inter-observer agreement (*p* > 96%).

**Conclusions:**

Our model achieved pathologist-level performance in diagnosing the presence or absence of coeliac disease from a representative set of duodenal biopsies, representing a significant advancement towards the adoption of machine learning in clinical practice. Additionally, it demonstrated strong generalisability, performing equally well on biopsies from a previously unseen hospital. We concluded that our model has the potential to revolutionise duodenal biopsy diagnosis by accurately identifying or ruling out coeliac disease, thereby significantly reducing the time required for pathologists to make a diagnosis.

## Introduction

The rise of digital pathology paired with decades of progress in computer vision has precipitated a rich variety of research in the automated detection of diseases and the creation of decision-support tools.^1^ In a time of unprecedented demand on pathologists^2–4^, such tools hold great appeal by promising to mitigate the time constraints associated with histological diagnosis.^5^ Moreover, advanced approaches offer the potential to improve diagnosis in developing nations, where shortages of pathologists are severe.^6,7^

While effort in this space has largely focused on the detection of cancer^8–12^, many other diseases remain overlooked. Although these conditions may be less severe, they significantly impact both patients’ quality of life and healthcare systems. Leveraging the power of artificial intelligence (AI) and computational methods to improve diagnosis of these conditions represents an excellent opportunity to address clear and unmet clinical needs.

One area in which modern diagnostic methods can excel and pioneer strong clinical precedents is the duodenum (first part of the small intestine). The majority of biopsies from this site are diagnosed as normal, while the most prevalent specific abnormality is coeliac disease (CD).^13^

In CD, ingestion of gluten (found in wheat, barley, and rye^14^) triggers an autoimmune response, causing symptoms including malabsorption, diarrhoea, weight loss, fatigue, anaemia, dermatitis herpetiformis (an itchy skin rash), and infertility.^15^ Symptom variation between patients makes accurate diagnosis more challenging, with asymptomatic patients still being at risk of long-term complications.^16^

### Current diagnosis and treatment

**Figure 1:**
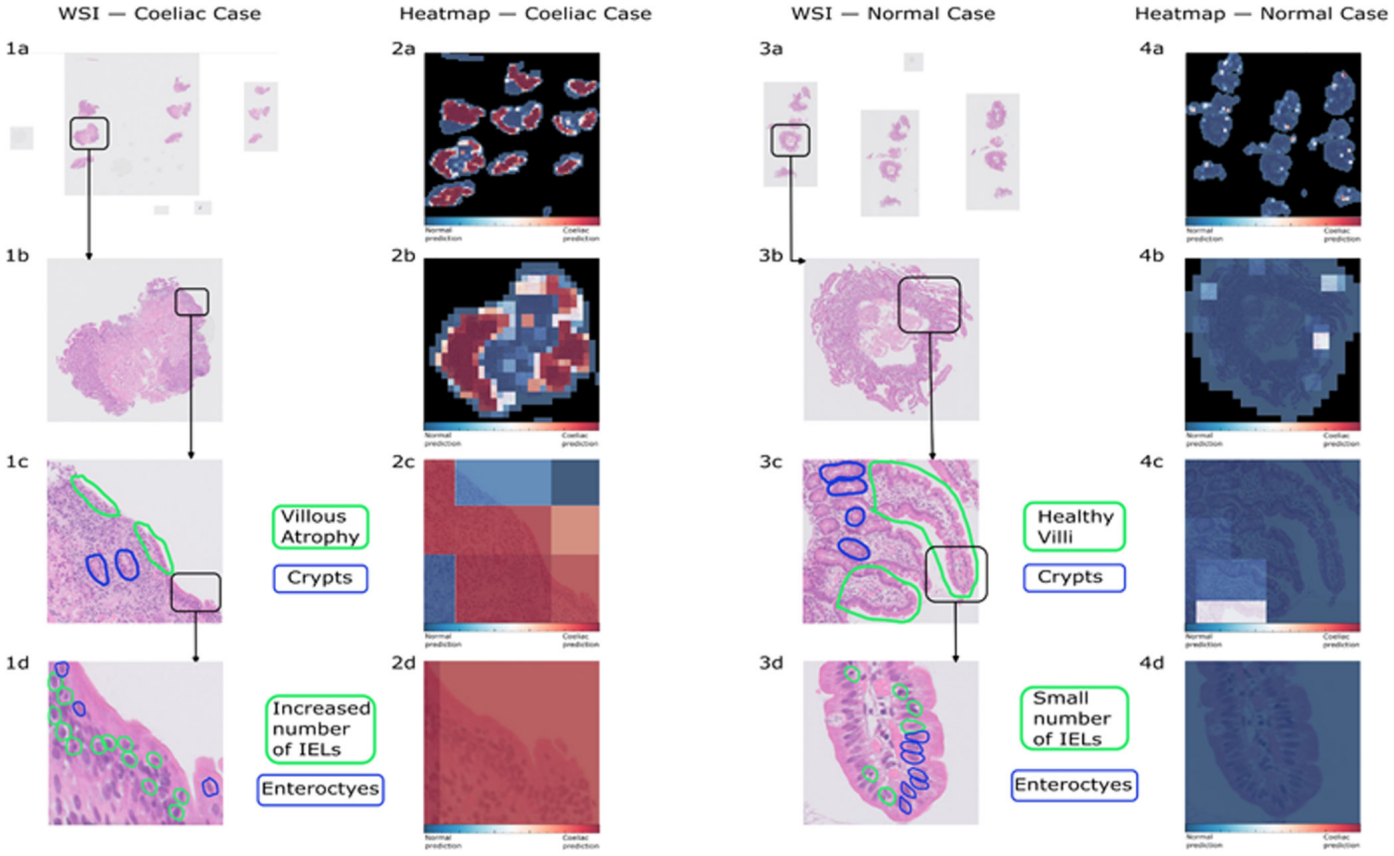
Visualisation of one of the cross-validation model's output. The first and third columns contain a CD and a normal WSI, respectively; the second and fourth column contain heatmaps of the same two case, with patches diagnosed by the model as CD highlighted in red and normal patches in blue. The first row shows the entire WSI and every following row has higher magnification sub-images. The third row clearly shows the difference between healthy villi (3c), diagnosed by the model as normal (4c), and villous atrophy (1c), where the villi are barely visible, a classic symptom of CD, which is also diagnosed as such by the model (2c). Moreover, one can clearly see crypts in the third row, however, unlike in some other CD cases, they appear normal in shape. In the fourth row we can clearly see individual enterocytes and intra-epithelial lymphocytes (IELs). Subfigure (1d) clearly shows an increased number of IELs, another common characteristic in coeliac disease cases. Patch (3d) has a much lower number of IELs, indicative of a lack of inflammation. We hypothesis that the villous blunting and raised IEL count cause the model to diagnose the patch as coeliac disease (2d), and the healthy-looking villi along with a low IEL count lead to the model’s normal diagnosis in (4d).

Although serological tests alone are sometimes used to diagnose coeliac disease^17,18^, the gold standard remains a duodenal biopsy. Pathologists diagnose coeliac disease by identifying characteristic histological changes in the duodenum, such as villous atrophy, crypt hyperplasia, and an increased number of intraepithelial lymphocytes^19^ (labelled in Figure 1).

Inter-observer agreement studies have shown that the concordance among pathologists when diagnosing coeliac disease can be as low as 70%^19–29^ due to the subjective nature of histological interpretation and the subtlety of the changes in some biopsies, highlighting the need for more objective and reliable diagnostic methods. The Marsh-Oberhuber classification is used by some pathologists to assess the severity of the changes in an attempt to increase objectivity and reproducibility of diagnosis, but it is largely a research tool.^30,31^

### Related research

A small number of studies have already investigated the automated detection of CD in H&E-stained whole-slide images (WSIs), but unlike this study, they have not been tested on a large, unselected test set.

Wei et al. divided WSIs into 224 × 224 pixel patches and trained a convolutional neural network to classify the patches achieving accuracies of 95.3%, 91.0%, and 89.2% for CD, normal tissue, and non-specific duodenitis, respectively.^32^ Limitations include the test set not being derived from an independent unseen source and their labelling of all patches from disease-containing WSIs as disease-positive, despite the significant possibility that not all patches may contain evidence of disease.

Sali et al. attempted to predict the Marsh–Oberhuber classification^30,31^ by using patches labelled with the Marsh–Oberhuber score of the biopsy they originated from.^33^ While this method is interesting, it only included 162 slides from 34 patients, and omitted class II (including only classes I, IIIa, IIIb, and IIIc) from the Marsh–Oberhuber scale.

Gruver et al. applied a machine learning approach to CD3 immunostained WSIs to count IELs and correlate them with the Marsh–Oberhuber classification.^34^ The lack of routine immunostaining for this biopsy type renders the translation of this research to the clinic more challenging than H&E-based methods.

In recent work, Denholm et al. demonstrated the utility of multiple instance learning in the automated detection of CD.^35^ Such weakly-supervised approaches are interesting because they circumvent the limitation of the labelling strategy used by Wei et al., allowing the localisation of predictions and therefore greater interpretability. The main limitation of the study by Denholm et al. is its limited applicability to a real-life clinical service, as it includes only unambiguous cases of CD and normal duodenal mucosa.

### Our contribution

**Figure 2:**
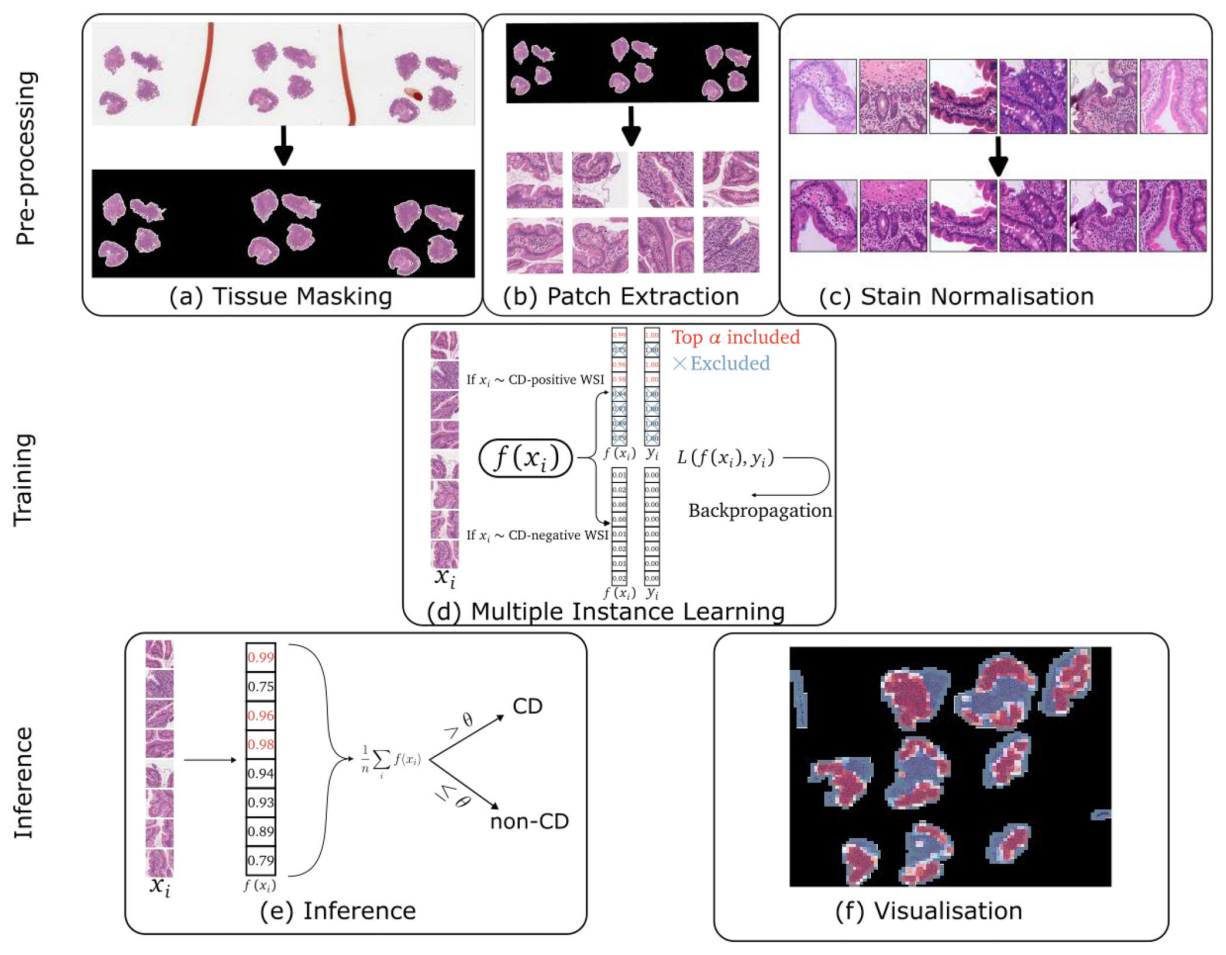
The five steps in our pipeline: (a) we obtain tissue masks separating the tissue from the background in low magnification overviews of WSIs; (b) we extract tissue-containing patches from the masked regions; (c) we apply stain normalisation (d) we train a machine learning classifier under a multiple instance learning paradigm; (e) we infer across all patches from a given WSI, map from patch-to slide-level predictions, and apply the threshold θ to make a diagnosis; (f) we provide a spatial context to the predictions by overlaying the patch-level predictions on the WSIs.

We present a deep-learning-based diagnostic classifier for CD that operates on histopathological image data. Our model has been comprehensively trained and tested on a large-scale, diverse dataset consisting of over 4,000 WSIs obtained from five different hospitals using five different scanners from four different companies. This dataset therefore encompassed a wide range of inter-laboratory and inter-scanner heterogeneity (see Figure 2 (c)). To the best of our knowledge, this is the largest and most diverse dataset of duodenal biopsies available, featuring representative real-world data. Our model achieved pathologist-level accuracy in diagnosing coeliac disease and generalised well to cases from unseen sources not included in the training data.

## Methods

### Whole-slide image preprocessing

To remove portions of WSIs containing no tissue and to remove confounding artefacts (pathologists’ pen marks, dark spots, and bounding boxes added by scanners, which can confuse any machine learning model^36^) we segmented the tissue-containing regions using Schreiber’s method^36^ (see Figure 2 (a)).

### Patch extraction

To manage the scale of the WSIs, we used QuPath^37^ to split the WSIs into patches of 256 × 256 pixels, at 10x magnification (≈ 1 *μ*m per pixel, with minor deviations between different scanners), using a sliding-window approach (applied convolutionally) with a stride of 128 pixels, resulting in a 50% overlap between adjacent patches. Patches with less than a 25% area-of-intersection with the tissue mask created with Schreiber’s method were discarded (see Figure 2 (b)). We chose 10x magnification as individual cells are clearly visible (see Figure S8, Supplementary Appendix I) and it reduces computational cost by a factor of 16 compared to using 40x magnification.

### Stain normalisation

Due to widespread inter-laboratory heterogeneity in staining protocols and digitisation artefacts from various commercial scanners, biopsies from different centres exhibit non-uniform colour statistics, thus inhibiting generalisability. We therefore used Macenko’s method for stain normalisation^38^, without any stain jittering, examples of which are shown in Figure 2 (c). Stain normalisation has been shown by numerous studies to significantly improve generalisation performance.^39-41^

### Model training

We trained the model using the multiple instance learning paradigm.^8,42,43^ Labelling every patch of a CD-positive WSI as CD-positive may lead to suboptimal results as many patches of a CD-positive biopsy may appear normal. To address this, we proposed a weakly-supervised approach to label the patches automatically.

During each training step, we first sampled a bag of 100 patches uniformly at random (with replacement) from a single WSI, due to the very large number of patches per slide. If the WSI had been diagnosed as CD-positive, we created proxy labels where the *α* = 20 “most positive” patches (as judged by the current version of the model) were labelled as positive and all other patches in the bag were discarded. If patches were sampled from a CD-negative slide, every patch was labelled as negative and included in the training step (see Figure 2 (d)).

We trained a total of five separate models using five-fold cross-validation, ensuring that the relative abundance of each class in the training set was preserved in each fold. Additionally, scans originating from the same block of tissue were always placed in the same fold. All five models were trained on data from all four hospitals using identical hyper-parameters, ensuring consistency in the training process.

### Experimental details

We implemented our code in PyTorch^44^, utilising a ResNet-18 architecture^45^ initialised with ImageNet-pretrained weights.^46^ The model was trained using the Adam optimiser^47^ with a constant learning rate and weight decay set to 1 × 10^−4^. As all patches within a batch come from the same WSI and are thus highly dependent, we employed instance normalisation instead of batch normalisation, with the affine parameter set to false. We further used the sigmoid activation function to ensure the model outputted a single normalised value between zero and one. The model was trained for 20 epochs using the binary cross-entropy loss function. For data augmentation, we applied random horizontal and vertical flips.

We justify the choice of every hyper-parameter in Supplementary Appendix F. Most hyper-parameters, including model architecture, number of training epochs, and the multiple instance learning parameters, are based on a thorough hyper-parameter optimization performed in Supplementary Appendix E.

### Model inference

At inference time, we applied the same WSI pre-processing, patch extraction, and stain normalisation steps as during training. We then ran the model independently on each patch and averaged the model output across all patches. If the mean model output exceeded a specified threshold, the model made a coeliac-positive diagnosis; otherwise, it made a negative diagnosis (see Figure 2 (e)). We determined the optimal threshold for each model to be the value between zero and one that maximises the difference between the true positive rate (TPR) and the false positive rate (FPR) on the validation set. Further details on the threshold computation, along with illustrations of the distributions of the raw model outputs, are provided in Supplementary Appendix G.

For testing the model on an independent test set from a previously unseen hospital, we used an ensemble model that takes the diagnosis from each of the five cross-validation models and returns the majority decision.^48^

### Performance metrics

We evaluated the performance of our method by calculating accuracy, sensitivity (also called recall), specificity, positive predictive value (PPV) (also called precision), negative predictive value (NPV), and the area under the receiver operating characteristic curve (ROC AUC).

We further compared our model’s performance to that of four independent pathologists on a subset of the unseen test data (30 images: ten normal, ten with coeliac disease, and ten with other diagnoses). The pathologists reviewed the WSIs using the Comparative Pathology Workbench (CPW), a digital image viewer developed at the University of Edinburgh.^49^ We calculated the mean average agreement, indicating the proportion of cases on which two diagnosticians agree and the Cohen’s kappa coefficient.^50^

### Data

We trained and tested our machine learning model on a diverse dataset (Table 1). Importantly, the test set cases originated from a source hospital distinct to the training and validation set samples. In a small subset of duodenal biopsy cases where the original histopathological diagnosis made by the originating hospital showed significant uncertainties, these cases were reviewed along with the associated clinical data (TTG, EMA, Hb, etc.) and where possible a more definitive diagnosis was provided by a referral expert consultant gastrointestinal histopathologist. All scans (and accompanying fully anonymised patient data) were obtained with full ethical approval (IRAS: 162057; PI: Prof. E. Soilleux).

## Results

### Cross-Validation

We trained our model on a diverse dataset of 3,383 WSIs of H&E-stained duodenal biopsies from four hospitals featuring five different WSI scanners along with their clinical diagnoses, using a five-fold cross-validation approach.

We evaluated the performance of the five cross-validation models by running the models on their respective hold-out validation sets. For each scan, we summarised the predictions by computing the mean of the patch-level predictions. We achieved ROC AUC values between 99.2% and 99.7% across the five different folds (Figure S1, Supplementary Appendix B).

To convert the model output into a final prediction, we used the thresholds computed for each model that maximise the difference between the true positive rate and the false positive rate on the validation set. Our models achieved a mean accuracy of 96.8%, with sensitivity and specificity values of 95.4% and 97.2%, PPV of 91.4%, and NPV of 98.5% (Table S1, Supplementary Appendix B).

### Different-Source Testing

We evaluated the generalisability of our models on a test set from a previously unseen source: the Western General Hospital in Edinburgh (Table S1, Supplementary Appendix B). We used an ensemble that returns the majority decision of the five cross-validation models. The model achieved an accuracy of 97.5%, and sensitivity and specificity values of 95.5% and 97.8%, respectively. These results demonstrate that our model generalised very well to cases from new hospitals.

Heatmaps representing the raw diagnostic predictions for each patch provided a visual representation of the model’s decision-making process (Figure 1; Figures S4-S7, Supplementary Appendix H). We found that as expected for most coeliac disease biopsies, the model classifies most patches around the edge of the tissue as coeliac disease. This is where pathologists focus on when making a diagnosis, as villous atrophy and a raised IEL count can be detected there (see Figure 1).

### Subgroup Testing

**Figure 3:**
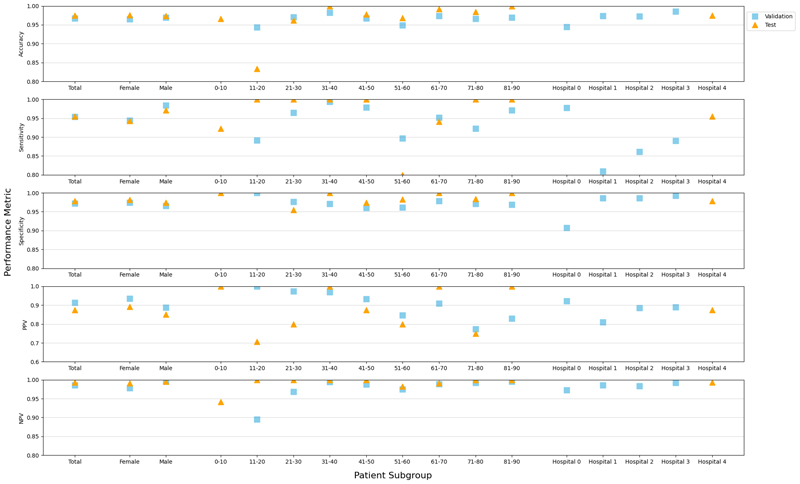
Validation and Test performance of our models broken down for each subgroup of patients based on age, sex, and hospital.

To evaluate potential biases in our model, we conducted a comprehensive analysis across various subgroups within our patient population. Figure 3 illustrates the validation and test performance of the model, stratified by age, sex, and hospital source. Our findings indicated that the model consistently achieved high accuracy across all subgroups, with performance metrics exceeding 94%, demonstrating its unbiased nature. The only exception was observed in the 10-19 year age group within the test set, where the accuracy is slightly lower, which may be caused by the limited sample size (n=29). Overall, our approach proved to be highly effective and equitable across diverse patient demographics.

### Inter-Observer Variability

**Figure 4:**
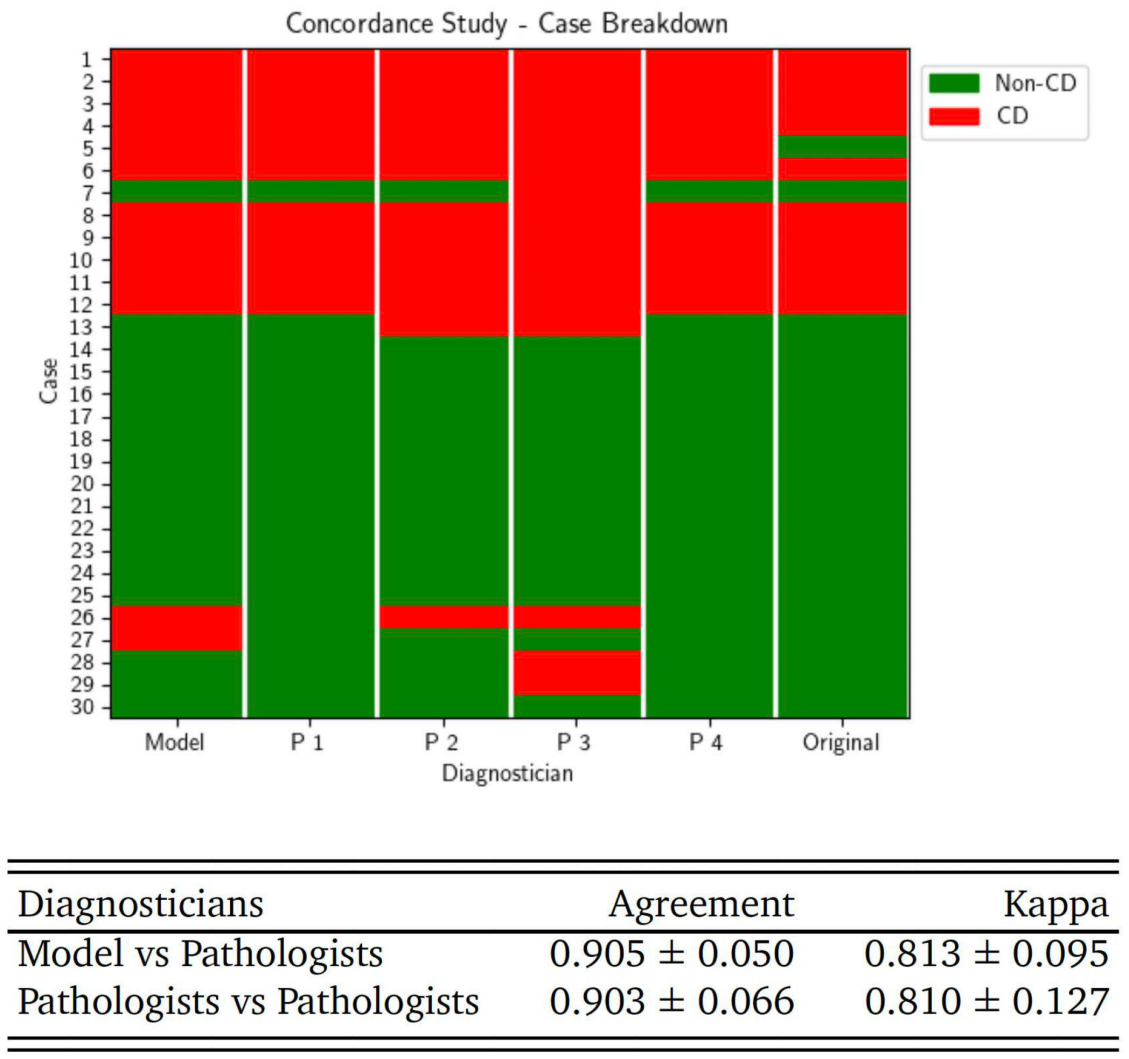
Concordance Study. Top: each row is a case, red is a CD diagnosis, green a non-CD diagnosis. The first column is our model. The next four columns are the pathologists, and the final column is the previous clinical diagnosis we received from the hospital. Bottom: the mean agreement between our model and each of the four pathologists is 90.5% which is comparable to the mean agreement between each pair of pathologists.

We analysed the agreement between the model and four pathologists on a subset of the test set. As we focused solely on the diagnosis of coeliac disease, we combined all *normal* and *other* diagnoses into a non-coeliac class, and all *coeliac* diagnoses into another. We observed a pair-wise mean agreement value between the four pathologists of 0.903 ± 0.066, with a corresponding kappa coefficient of 0.81 ± 0.127. The average pair-wise agreement between the machine learning model and each pathologist was nearly identical, at 0.905 ± 0.050, with a corresponding kappa coefficient of 0.813 ± 0.095 (Figure 4; Tables S4 and S5, Supplementary Appendix C).

We conducted an independent samples t-test, assuming unequal variances (Welch’s t-test), to compare the pairwise agreement values between the model and pathologists with the pairwise agreement values among pathologists. The pairwise agreement analysis yielded a t-statistic of 0.045 and a p-value of 0.965 with a kappa metric t-statistic of 0.036 and a p-value of 0.973. These high p-values indicate that there is no statistically significant difference between the pairwise agreement values of the model with the pathologists and those among the pathologists themselves.

We concluded that our model achieves pathologist-level performance in diagnosing coeliac disease.

## Discussion

We present a machine learning model that achieves pathologist-level diagnosis of coeliac disease. This represents a crucial step towards the clinical implementation of machine-learning-assisted pathology for diagnosing coeliac disease. This achievement is significant for several reasons: i) there is a critical global shortage of pathologists in both developing and developed countries, often resulting in long delays for patient diagnoses^51,52^, ii) coeliac disease, with an estimated global prevalence of at least 1%, is the most common pathological diagnosis for duodenal biopsies aside from normal findings^13^, iii) numerous studies have shown low concordance between pathologists when diagnosing coeliac disease.^19–29^

Our machine learning model, which achieved human-level performance, demonstrates significant potential in addressing these critical issues. It achieved an accuracy, sensitivity, and specificity exceeding 95% on an independent test set composed of cases from sources not included in the training and validation data. To the best of our knowledge, this is the first study to show AI achieving human-level performance in coeliac disease diagnosis on a genuine, multi-centre, clinically-representative cohort of patient samples. This level of generalisability is crucial for deploying AI models in real-world clinical environments, where variability in staining protocols and scanner technology can significantly impact diagnostic accuracy. We also show that our model worked equally well for patients of all sexes and ages above 19 years.

Our concordance study further demonstrates that the model’s performance is statistically indistinguishable from that of human pathologists. This concordance underscores the potential of AI to automatically diagnose coeliac disease.

Heatmaps show that, like pathologists, our model mostly classifies coeliac disease around the edges of the biopsy. However, we also show in the Supplementary Appendix that some of the more central patches are still classified as coeliac disease, showing the potential for the model to outperform pathologists by finding patterns in areas of the biopsy that most pathologists focus less on. Future work should focus on using attention-based multiple-instance learning algorithms, such as CLAM^53^, as they have the potential to improve model accuracy, whilst also providing informative heatmaps that improve interpretability of the model’s output. We do, however, note that — unlike other conditions such as tumours or the presence of parasites — coeliac disease cannot be localised quite in the same way, so heatmaps are inherently less informative than they are for other conditions.

The main known limitation of this work, and much of the work on coeliac disease, is the accuracy of the ground truth due to known disagreements between pathologists when diagnosing coeliac disease. These disagreements are more frequent than for many other areas of pathology.^54^ The concordance study we present in this work aims to mitigate the limitations of the dataset, but only includes 30 cases and does not provide perfect ground truth diagnoses either.

## Conclusion

In conclusion, we have presented the first machine learning diagnostic tool for coeliac disease that demonstrates human-level accuracy on duodenal biopsies from a hospital different from its training data. We demonstrate that the concordance between the model and pathologists is similar to the agreement among pathologists. Furthermore, we demonstrate that our model is unbiased and performs equally well for male and female patients of all ages over 19 years.

## Supplementary Material

Supplementary Materials

## Figures and Tables

**Table 1:** Details of our dataset, including training data from four different hospitals and five different scanners. Sex and age data were not available for the biopsies from North Tees, and seven cases from Addenbrookes had missing sex data. Each scan from the same patient was treated as a separate case, meaning a single patient may appear multiple times across different categories.

			Training Data			Testing Data	
		Coeliac (*n* = 756)	Normal (*n* = 2083)	‘Other’ (*n* = 544)	Coelia (*n* = 88)	Normal (*n* = 525)	‘Other’ (*n* = 31)
Source	Addenbrookes	73	779	256	-	-	-
	North Tees	21	246	32	-	-	-
	Heartlands	590	518	209	-	-	-
	Glasgow	72	540	47	-	-	-
	Edinburgh	-	-	-	88	525	31
Scanner	Leica Aperio AT2	253	950	321	-	-	-
	Hamamatsu 210	21	246	32	-	-	-
	Roche Ventana iScan HT	230	176	79	-	-	-
	Hamamatsu	180	171	65	-	-	-
	Nanozoomer XR						
	Philips IntelliSite	72	540	47	88	525	31
	Ultra Fast Scanner						
Sex	Female	486	1063	287	53	313	12
	Male	247	769	225	35	212	19
Age	median	44	64	59	47	62	41
	10 - 19	26	19	4	12	7	10
	20 - 29	122	107	35	5	18	4
	30 - 39	148	138	51	8	17	0
	40 - 49	151	222	74	13	68	3
	50 - 59	81	286	100	9	111	4
	60 - 69	111	385	94	19	109	4
	70 - 79	57	443	86	6	121	3
	80 - 89	37	217	65	4	57	2
	90 - 99	0	15	3	0	2	0

## Description

A machine learning model that diagnoses coeliac disease from duodenal biopsy images demonstrates strong generalizability across multiple hospitals and has the potential to enhance diagnostic efficiency and reliability in clinical practice.
