# Supplementary material for: Machine Learning Achieves Pathologist-Level Coeliac Disease Diagnosis
Source: NEJM AI. Author manuscript; Available in PMC 2025 May 28. (PMC7617718; doi:10.1056/AIoa2400738)
Supplement: Supplementary Materials [file EMS204246-supplement-Supplementary_Materials.pdf]

Supplementary Materials for Machine Learning Achieves Pathologist-Level Coeliac Disease Diagnosis

F. Jaeckle,[1,2,*] J. Denholm,[1,2,3,†] B. Schreiber,[1,3,‡] S. C. Evans,[1] M. N. Wicks,[4]
J. Y. H. Chan,[5] A. C. Bateman,[6] S. Natu,[7] M. J. Arends,[4] and E. Soilleux[1,2]

[1]*Department of Pathology, University of Cambridge, Tennis Court Road, CB2 1QP, Cambridge, England, UK.*
[2]*Lyzeum Ltd, Cambridge, CB1 2LA, England, UK.*
[3]*Department of Applied Mathematics and Theoretical Physics, University of Cambridge, Wilberforce Road, CB3 0WA, Cambridge, England, UK.*
[4]*Edinburgh Pathology & Centre for Comparative Pathology, Institute of Genetics & Cancer, University of Edinburgh, Crewe Road, Edinburgh EH4 2XR, UK*
[5]*Cambridge University Hospitals NHS Foundation Trust, Cambridge, UK*
[6]*University Hospital Southampton NHS Foundation Trust, Southampton, UK*
[7]*University Hospital of North Tees, North Tees and Hartlepool NHS Foundation Trust, Hardwick, Stockton on Tees, England, UK.*

(Dated: October 4, 2024)

1. Appendix A describes how we collected our dataset in more detail.
2. Appendix B includes a more detailed analysis of the experimental results.
3. Appendix C focuses on the agreement study summarised in the main paper.
4. Appendix D includes two further sets of experiments. The former trains four cross-validation models on three centres and validates them on the remaining fourth. The latter trains four cross-validation models each on only one centre and validates them on the remaining three.
5. Appendix E performs a comprehensive hyper-parameter analysis. We compare different model architectures (including a pre-trained CTransPath model), number of epochs, and MIL parameters.
6. Appendix F includes a list of every hyper-parameter used in this study including a justification for the chosen values.
7. Appendix G describes in more detail how we generate the threshold values that turn the model outputs into a diagnosis and also includes a detailed analysis of the distribution of raw model outputs.
8. Appendix H focuses on the explainabiltiy of our method and contains further heatmaps comparing the output of the five cross-validation models on four coeliac cases from the test set.
9. Appendix I contains a Figure containing 8 example patches to demonstrate the size and magnification of the input images for the model.

Appendix A: Dataset

We now explain in a bit more detail how the dataset was collected. As described in Table I. in the main paper, we collected real world biopsies from five different UK hospitals. For some of the hospitals we collected all biopsies in a specified time frame, and others we first collected unselected biopsies before adding more coeliac disease cases randomly selected from a different time period to enrich our dataset.

For the cases from Addenbrookes hospital we picked all duodenal biopsies diagnosed over a time frame of several months. For the cases from North Tees hospital we also collected all cases for a given time frame. For images from Edinburgh hospital we first picked all duodenal biopsies for a given time frame and then added a few more coeliac disease and other cases that were diagnosed earlier that year. For the images from Glasgow we first picked all duodenal biopsies for a given time frame and then added a few more coeliac cases from other months. The WSIs from Heartlands were picked by a pathologists and include a higher number of coeliac disease cases than normal.

* fj286@cam.ac.uk; Equal contribution
† jd949@cam.ac.uk; Equal contribution
‡ Equal contribution

Appendix B: Test Results - Further analysis

We provide a more detailed analysis of the results presented in the main paper. We provide ROC and AUC curves of the validation set in Figure S1. In Table S1 we summarised the results of the five cross-validation models on the validation set, as well as those of the ensemble on the test set. In table Table S2 we present the results of each cross-validation model on the test set. We further break down the performance of the ensemble my summarising the agreement between the five cross-validation models in Table S3.

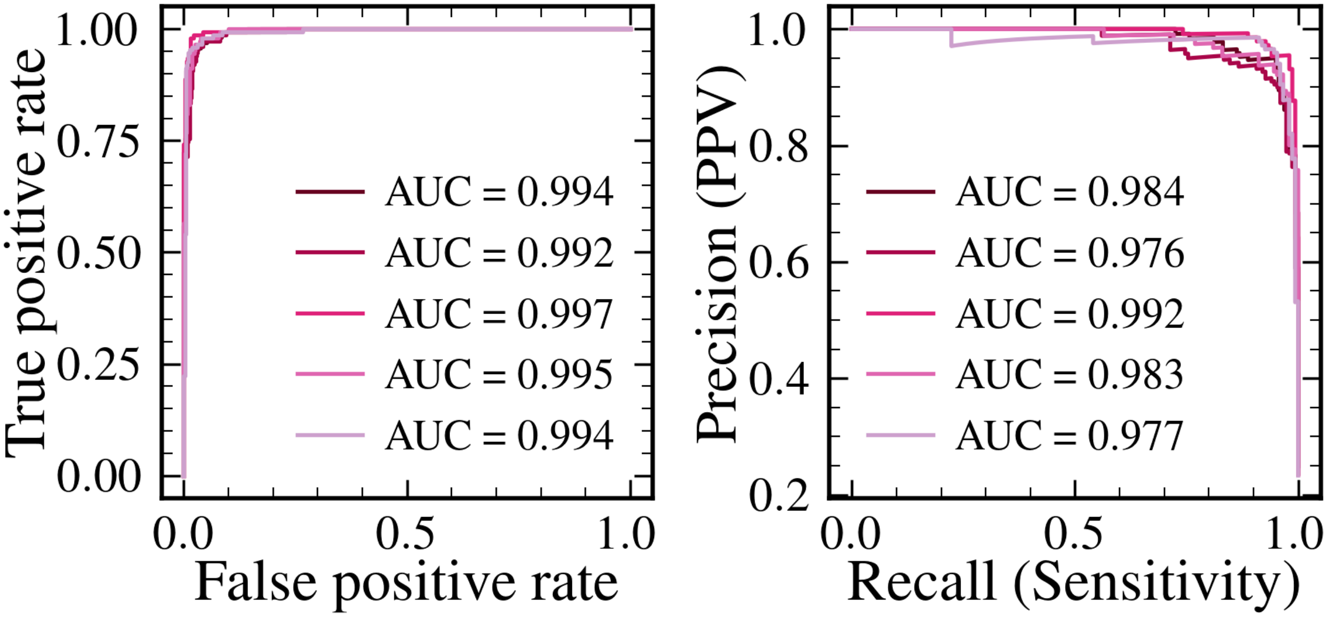

FIG. S1. ROC and PR curves for each cross-validation fold. All five cross-validation models achievedan area under the ROC curve of over 99% and AUC values of the PR curve of over 97% on the validation set.

TABLE S1. Main results. We first evaluated our machine learning model in a five-fold cross-validation setting on the validation dataset. We achieved a mean accuracy of 96.8% and sensitivity and specificity values of over 95%. We then test our main model, an ensemble consisting of the five cross-validation models, on the different-source test dataset. The model has not been trained on any cases from this hospital. The model achieves an accuracy of 97.5% and sensitivity and specificity values exceeding 95% on the test set.

	Fold	Accuracy	Sensitivity	Specificity	PPV	NPV	ROC-AUC
Validation	0	0.955	0.944	0.959	0.883	0.981	0.994
	1	0.959	0.927	0.969	0.903	0.977	0.992
	2	0.983	0.980	0.984	0.949	0.994	0.997
	3	0.969	0.966	0.970	0.910	0.989	0.995
	4	0.972	0.953	0.977	0.928	0.986	0.994
	Mean	0.968	0.954	0.972	0.914	0.985	0.994
	Std	0.011	0.020	0.010	0.025	0.007	0.002
Test	Model	0.975	0.955	0.978	0.875	0.993	-

TABLE S2. We evaluated the five cross-validation models on the test set.

Fold	Accuracy	Sensitivity	Specificity	PPV	NPV	ROC-AUC
0	0.967	0.977	0.966	0.819	0.996	0.994
1	0.974	0.955	0.977	0.866	0.993	0.995
2	0.961	0.773	0.991	0.932	0.965	0.996
3	0.696	1.000	0.647	0.310	1.000	0.990
4	0.966	0.875	0.980	0.875	0.980	0.995
Mean	0.913	0.916	0.912	0.760	0.987	0.994
Std	0.121	0.093	0.148	0.255	0.014	0.002

TABLE S3. Breakdown of the ensemble member's diagnosis. Numbers in bold lead to correct classifications by the ensemble. We separate the cases into three categories (Coeliac, Normal, Other), for a more detailed analysis, but the model only outputs Coeliac or not Coeliac. As we have 5 models, the ensemble diagnoses Coeliac if and only if at least 3 of models classify the case as coeliac.

	Number of models that diagnose coeliac					
	0	1	2	3	4	5
Coeliac	0	1	3	**5**	**14**	**65**
Normal	**353**	**167**	**3**	1	1	0
Other	**7**	**9**	**5**	0	5	5

Appendix C: Concordance Study - Further Analysis

We further analyse the agreement between the model and the four pathologists on the subset of the test set. In table S4 we summarised the agreement and kappa value for every pair of diagnosticians. In table S5 we summarised the agreement for each diagnostician with every other diagnostician.

TABLE S4. Concordance. Analyse the agreement of each pairs of diagnostician

Diagnostician 1	Diagnostician 2	agreement	kappa
ML Model	Pathologist 1	0.93	0.86
ML Model	Pathologist 2	0.93	0.86
ML Model	Pathologist 3	0.83	0.67
ML Model	Pathologist 4	0.93	0.86
Pathologist 1	Pathologist 2	0.93	0.86
Pathologist 1	Pathologist 3	0.83	0.67
Pathologist 1	Pathologist 4	1.00	1.00
Pathologist 2	Pathologist 3	0.90	0.80
Pathologist 2	Pathologist 4	0.93	0.86
Pathologist 3	Pathologist 4	0.83	0.67

TABLE S5. Concordance. Average Agreement of each diagnostician with the rest of the diagnosticians.

Diagnostician	agreement	kappa
ML Model	0.905	0.812
Pathologist 1	0.922	0.848
Pathologist 2	0.923	0.845
Pathologist 3	0.848	0.703
Pathologist 4	0.923	0.847

Appendix D: Additional Experiments

1. Cross-Validation by source

We now run cross-validation experiments where instead of randomly diving our training dataset into 5 folds, we define each fold to be made up of all cases from one specific centre and train the corresponding model on all cases from the remaining three centres. Table S6 includes a 4-cv experiment where we train the model using the identical set-up to the main paper. We report a mean accuracy of 93%, varying from 91.5% to 96.6%. As expected, theses accuracies are lower than the one reported in Table S1 where we models have been trained and evaluated on cases from all four hospitals.

Sources	Accuracy	Sensitivity	Specificity	PPV	NPV	ROC AUC
Addenbrookes	0.966	0.945	0.967	0.670	0.996	0.993
North Tees	0.916	0.952	0.914	0.455	0.996	0.987
Glasgow	0.915	0.931	0.913	0.568	0.991	0.984
Heartlands	0.920	0.908	0.932	0.939	0.899	0.975
mean	0.929	0.934	0.932	0.658	0.971	0.985
std	0.024	0.019	0.025	0.207	0.047	0.007

TABLE S6. Cross validation by source experiments. We train four models, each on three of the four centres and validate them on the centre specified under the "Sources" column. All four models achieve good generalisation performance, however, they perform slightly worse than the 5-cross-validation models that have been trained on all 4 centres summarised in Table B.

2. One Centre Training

To highlight the importance of using a comprehensive dataset, we trained four models on only one centre each and evaluated the models on the three remaining centres used for training and validation. The models trained on Addenbrookes, Heartlands, Glasgow, and North Tees have been trained for 20, 20, 35, and 50 epochs, respectively, due to the different size of the training sets. The results are summarised in Table S7. We conclude that the performances of all of the four trained models are significantly worse than the models trained on three centres, showing the importance of a large and diverse dataset.

Training Source	Validation	Accuracy	Sensitivity	Specificity	PPV	NPV	ROC-AUC
Addenbrookes	Heartlands	0.870	0.851	0.892	0.900	0.840	0.953
Addenbrookes	North Tees	0.836	0.952	0.827	0.294	0.996	0.965
Addenbrookes	Glasgow	0.883	0.875	0.884	0.481	0.983	0.949
Glasgow	Heartlands	0.773	0.653	0.911	0.893	0.697	0.901
Glasgow	North Tees	0.779	1.000	0.763	0.241	1.000	0.965
Glasgow	Addenbrookes	0.686	1.000	0.664	0.173	1.000	0.978
Heartlands	North Tees	0.870	0.857	0.871	0.333	0.988	0.956
Heartlands	Glasgow	0.930	0.917	0.932	0.623	0.989	0.977
Heartlands	Addenbrookes	0.945	0.918	0.947	0.549	0.994	0.985
North Tees	Heartlands	0.839	0.751	0.940	0.935	0.768	0.935
North Tees	Glasgow	0.926	0.375	0.993	0.871	0.928	0.934
North Tees	Addenbrookes	0.728	1.000	0.709	0.195	1.000	0.977

TABLE S7. Models trained on only one source.

Appendix E: Hyper-paramter Optimisation

1. CTransPath pretrained encoder

We use the CTransPath encoder as the feature extractor, fix it's weights and train a linear classifier to convert the 768-dimensional feature vectors into a coeliac prediction. We use a the adam optimiser with a learning rate of 0.0002 and a weight decay of 0.00001 as suggested by the authors for the WSI classification fine-tuning task. We don't use stain-normalisation but instead use the same data normalisation procedure as in the original paper (mean=[0.485, 0.456, 0.406],std=[0.229, 0.224, 0.225])). We further randomly crop the 256x256 sized patches to 224x224 to match the image size of the pretrained CTransPath model. The averaged results over the four models for each hyper-paramter setting are summarised in table S8. The results are significantly worse than the ones achieved by our main model. The detailed results of each individual cross-validation model can be found in Table S11.

Encoder	Pre-training	Epochs	Accuracy	Sensitivity	Specificity	PPV	NPV	ROC-AUC
ResNet18	ImageNet	10	0.921	0.911	0.923	0.647	0.962	0.975
ResNet18	ImageNet	20	0.929	0.934	0.932	0.658	0.971	0.985
ResNet34	ImageNet	10	0.889	0.898	0.883	0.561	0.954	0.961
CTransPath	CTransPath	10	0.735	0.765	0.693	0.374	0.905	0.809
CTransPath	CTransPath	20	0.806	0.755	0.803	0.428	0.903	0.862

TABLE S8. Hyper-parameter study focusing on the model architecture, pre-trained weights, and the number of epochs used during training. We note that ResNet18 outperforms ResNet34 as well as CTransPath.

2. Multiple Instance Learning parameters

We now optimise over the multiple instance learning hyper-parameters. We note that increasing or decreasing the MIL bag size or the number of training epochs achieves the same effect. We therefore fixed the bag size at 100 patches and optimised the number of epochs instead. We perform a hyper-parameter optimisation analysis for the alpha and beta parameters described in the main paper. We train four cross-validation models for 10 epochs, each on cases from three centresand valuate them on the respective remaining centre. We illustrate the main results averaged over the four runs in Table S9. We note that increasing the beta parameter from 0 does not improve performance. We further find the optimal alpha parameter to be around 40. The detailed results of each individual cross-validation model can be found in Table S11.

As alpha=40 get optimal performance we train a further five cross-validation models on random subsets of the training centre, including cases from all four cases (like in the main paper) for 20 epochs. The results are summarised in Table S10. In this setting alpha=20 achieves better performance. We thus conclude that the optimal value for the alpha hyper-paramter may depend on the exact training and validation scenario. We fix alpha=20 but encourage future work to focus on a more detailed review of the optimal MIL parameters.

Alpha	Beta	Accuracy	Sensitivity	Specificity	PPV	NPV	ROC-AUC
20	0	0.921	0.911	0.923	0.647	0.962	0.975
40	0	0.955	0.927	0.955	0.762	0.975	0.986
75	0	0.941	0.930	0.941	0.665	0.982	0.982
100	0	0.903	0.928	0.900	0.590	0.976	0.976
20	20	0.925	0.892	0.927	0.675	0.957	0.976

TABLE S9. Hyper-parameter study focusing on MIL parameters. We trained four Resnet18 model for 10 epochs each on three of the four centres and validated them on the fourth. The table includes the average performance over the four runs.

Alpha	Beta	Accuracy	Sensitivity	Specificity	PPV	NPV	ROC-AUC
20	0	0.968	0.954	0.972	0.914	0.985	0.994
40	0	0.959	0.958	0.960	0.883	0.986	0.994

TABLE S10. Hyper-parameter study focusing on MIL parameters. We trained five Resnet18 model for 20 epochs each on a random subet of cases from all four centres. The table includes the average performance over the four runs.

Epochs	Encoder	Alpha	Beta	Sources	Accuracy	Sensitivity	Specificity	PPV	NPV	ROC AUC
10	ResNet18	20	0	Addenbrookes	0.960	0.932	0.962	0.636	0.995	0.990
10	ResNet18	20	0	North Tees	0.933	0.952	0.932	0.513	0.996	0.988
10	ResNet18	20	0	Glasgow	0.904	0.875	0.908	0.538	0.983	0.958
10	ResNet18	20	0	Heartlands	0.888	0.886	0.890	0.902	0.873	0.962
10	ResNet18	20	0	mean	0.921	0.911	0.923	0.647	0.962	0.975
10	ResNet18	20	0	std	0.032	0.037	0.031	0.178	0.059	0.017
20	ResNet18	20	0	Addenbrookes	0.966	0.945	0.967	0.670	0.996	0.993
20	ResNet18	20	0	North Tees	0.916	0.952	0.914	0.455	0.996	0.987
20	ResNet18	20	0	Glasgow	0.915	0.931	0.913	0.568	0.991	0.984
20	ResNet18	20	0	Heartlands	0.920	0.908	0.932	0.939	0.899	0.975
20	ResNet18	20	0	mean	0.929	0.934	0.932	0.658	0.971	0.985
20	ResNet18	20	0	std	0.024	0.019	0.025	0.207	0.047	0.007
10	ResNet34	20	0	Addenbrookes	0.922	0.959	0.920	0.458	0.997	0.980
10	ResNet34	20	0	North Tees	0.936	0.905	0.939	0.528	0.992	0.984
10	ResNet34	20	0	Glasgow	0.845	0.861	0.843	0.403	0.980	0.943
10	ResNet34	20	0	Heartlands	0.850	0.866	0.832	0.855	0.845	0.937
10	ResNet34	20	0	mean	0.889	0.898	0.883	0.561	0.954	0.961
10	ResNet34	20	0	std	0.048	0.045	0.054	0.203	0.073	0.025
10	CTransPath	20	0	Addenbrookes	0.882	0.932	0.878	0.351	0.995	0.970
10	CTransPath	20	0	North Tees	0.528	0.524	0.529	0.077	0.936	0.608
10	CTransPath	20	0	Glasgow	0.856	0.778	0.865	0.415	0.969	0.899
10	CTransPath	20	0	Heartlands	0.674	0.829	0.498	0.653	0.719	0.758
10	CTransPath	20	0	mean	0.735	0.765	0.693	0.374	0.905	0.809
10	CTransPath	20	0	std	0.166	0.173	0.207	0.237	0.126	0.160
20	CTransPath	20	0	Addenbrookes	0.870	0.959	0.864	0.332	0.997	0.972
20	CTransPath	20	0	North Tees	0.756	0.524	0.773	0.149	0.956	0.758
20	CTransPath	20	0	Glasgow	0.897	0.806	0.908	0.518	0.974	0.925
20	CTransPath	20	0	Heartlands	0.701	0.731	0.668	0.715	0.685	0.790
20	CTransPath	20	0	mean	0.806	0.755	0.803	0.428	0.903	0.862
20	CTransPath	20	0	std	0.093	0.181	0.106	0.243	0.146	0.103
10	ResNet18	20	20	Addenbrookes	0.960	0.918	0.963	0.638	0.994	0.989
10	ResNet18	20	20	North Tees	0.923	0.952	0.921	0.476	0.996	0.986
10	ResNet18	20	20	Glasgow	0.942	0.819	0.957	0.702	0.977	0.976
10	ResNet18	20	20	Heartlands	0.873	0.878	0.867	0.882	0.862	0.953
10	ResNet18	20	20	mean	0.925	0.892	0.927	0.675	0.957	0.976
10	ResNet18	20	20	std	0.038	0.057	0.044	0.168	0.064	0.016
10	ResNet18	40	0	Addenbrookes	0.952	0.932	0.954	0.586	0.995	0.990
10	ResNet18	40	0	North Tees	0.973	0.905	0.978	0.760	0.993	0.987
10	ResNet18	40	0	Glasgow	0.926	0.903	0.928	0.607	0.987	0.975
10	ResNet18	40	0	Heartlands	0.940	0.946	0.932	0.941	0.938	0.980
10	ResNet18	40	0	mean	0.948	0.921	0.948	0.724	0.978	0.983
10	ResNet18	40	0	std	0.020	0.021	0.023	0.164	0.027	0.007
10	ResNet18	75	0	Addenbrookes	0.928	0.932	0.928	0.476	0.995	0.982
10	ResNet18	75	0	North Tees	0.950	0.952	0.950	0.588	0.996	0.990
10	ResNet18	75	0	Glasgow	0.933	0.875	0.940	0.643	0.984	0.968
10	ResNet18	75	0	Heartlands	0.953	0.959	0.946	0.953	0.953	0.988
10	ResNet18	75	0	mean	0.941	0.930	0.941	0.665	0.982	0.982
10	ResNet18	75	0	std	0.012	0.038	0.010	0.204	0.020	0.010
10	ResNet18	100	0	Addenbrookes	0.921	0.918	0.921	0.450	0.994	0.978
10	ResNet18	100	0	North Tees	0.957	0.952	0.957	0.625	0.996	0.990
10	ResNet18	100	0	Glasgow	0.803	0.903	0.790	0.346	0.985	0.952
10	ResNet18	100	0	Heartlands	0.934	0.937	0.931	0.939	0.929	0.984
10	ResNet18	100	0	mean	0.903	0.928	0.900	0.590	0.976	0.976
10	ResNet18	100	0	std	0.069	0.022	0.074	0.260	0.032	0.017

TABLE S11. Hyper-paramter optimisation. Each model is trained on 3 centres and validated on the remaining centre specified in the "Source" column.

Appendix F: Hyper-parameter Explanation

Finally, we explain the reasoning behind chosing the hyper-parameter outlined above in Table S12. For some of the parameters we picked values based on hyper-parameter optimisation results described above in table S8. For the remaining parameters we chose values inspired by other published work.

Hyper-parameter	Value	Reasoning
Learning rate	1e-4	Common learning rate value for ResNet18 models. We further observe that training is stable (indication that the learning rate is not too high), but increasing the number of epochs from 10 to 20, barely improves performance (indicates that the learning rate is not too low).
Weight decay	1e-4	Common learning rate value used for training ResNet18 models. Models generalise well
Type of optimiser	Adam	Adam is a very commonly used optimiser for ResNet models. We chose it because of its fast convergence rate and robustness to suboptimal hyper-parameter choices. Future work could try using SGD instead as it often leads to better generalization performance if the hyper-parameters are tuned well [1].
Number of epochs	20	We noticed only a very small improvement when trained for 20 epochs compared to 10 epochs (see Table S8), so no further increases in epochs were tested
Network architecture	ResNet18	We chose a small network to make inference as cheap as possible, improving the chances that the model would be economical enough to be used in practice. As shown in table S8, using the CTransPath model with a fine-tuned classifier did not improve performance.
Pre-trained weights	ImageNet	The most common publicly available pre-trained weights. Future work could focus on pre-training in a self-supervised manner on a large unlabelled pathology dataset, however, as we have a reasonable large training dataset, pre-training is not as essential.
Network Normalisation	Instance normalisation	We use instance normalisation rather than the more frequently used batch-normalisation, as the patches that make up a batch are not independent as they come from the same WSI.
Stain normalisation	True	We use stain normalisation as many studies have shown stain normalisation to improve generalisation performance. (Schreiber *et al.* [2] who performed a detailed analysis of the impact of stain normalisation and stain jittering on the generalisation of neural networks to whole slide images from different scanners. They highlighted a significant boost to generalizability by using stain normalization. Similarly, Ciompi *et al.* [3] showed an improvement in accuracy of AI diagnosis of rectal cancer of 20% when using stain normalisation, and Salvi *et al.* [4] found stain normalisation improving accuracy by 19% and 18% for AI diagnosis of prostate cancer and breast cancer, respectively.)
Magnification	10x	We chose 10x magnification as you can still see individual cells as well as macro-features in the biopsies. Further increasing the magnification, would make the model more expensive to run and thus decrease the likelihood of it being cheap enough to be used in practice. Furthermore, 10x magnification has also been used by other work on using AI for coeliac disease diagnosis [2, 5]. Other work on using AI on coeliac disease have used 20x magnification [6] and [7, 8], so future work should compare both the speed and accuracy of running ML algorithms on different types of magnification.
Patch Size	256	We picked a patch size used by [5]. However, future work could analyse the effect of changing the patch size on the accuracy and generalisability of the model.
Patch Stride	128	Picking a stride that is half the value of the patch width and height ensures that no parts of the tissue is only contained on the outside edges of a patch. This is important as it is more difficult to make sense of the edges of a patch.
Patch Tissue overlap threshold	0.25	This ensures that every patch contains at least 25% tissue. Different values should be tested as part of future work.
MIL bag size	100	Increasing or decreasing the MIL bag size or the number of training epochs achieves the same effect. We therefore fixed the bag size at 100 patches and optimised the number of epochs.
MIL alpha parameter	20	As seen in Tables S9 and S10 the optimal alpha parameter depends on the exact training scenario. We set alpha=20 as it gives optimal performance in the 5-cv setting, where we train on data from four hospitals.
MIL beta parameter	0	As seen in Table S9 increasing the beta parameter, does not improve performance, so we keep it at 0.

TABLE S12. We summarise the hyper-parameters used to train and infer on our models and give justification for the chosen values.

Appendix G: Thresholding

In order to turn the raw model output into a diagnosis we need to create a threshold. In order to maximise performance and as each cross-validation model is unique, we compute a separate threshold for each cross-validation model. It would not be fair to first compute a threshold on the validation dataset and then report the validation performance using that threshold. Therefore, we divide the validation set for each model into two halves. We first computed the optimal threshold on the first half and then evaluated the performance on the second half before computing the best threshold on the second half of the validation set and evaluating the model on the first half. This way no case is used for both calculating a threshold and for evaluation using the same threshold. We note that the random seed used to divide the validation dataset in two halves will impact both the two resulting thresholds and the accuracy. We summarised this process in table S13. For several folds the threshold for the two divisions varies significantly which shows that having a larger validation set would be beneficial.

When we evaluated the model on the test set, we recalculate the threshold on the entire validation set, rather than on just one half.

TABLE S13. Evaluation of the five cross-validation models on the validation set. We split each validation set into two divisions and compute the optimal threshold on one division and then evaluated it on the other division to make sure that no image is used for both computation of the threshold value and to report any performance metrics.

Fold	Division	Accuracy	Sensitivity	Specificity	PPV	NPV	ROC-AUC	Threshold
0	0	0.969	0.925	0.983	0.949	0.975	0.994	0.207
0	1	0.941	0.963	0.934	0.828	0.987	0.994	0.156
1	0	0.962	0.853	0.996	0.985	0.957	0.993	0.227
1	1	0.956	1.000	0.942	0.843	1.000	0.994	0.180
2	0	0.984	0.987	0.984	0.949	0.996	0.997	0.131
2	1	0.981	0.973	0.984	0.948	0.992	0.998	0.141
3	0	0.977	0.986	0.974	0.924	0.996	0.996	0.146
3	1	0.961	0.945	0.966	0.896	0.983	0.993	0.179
4	0	0.969	0.986	0.963	0.890	0.996	0.995	0.085
4	1	0.975	0.919	0.992	0.971	0.976	0.993	0.112
mean		0.968	0.954	0.972	0.918	0.986	0.995	0.157
std		0.013	0.045	0.021	0.053	0.013	0.002	0.043

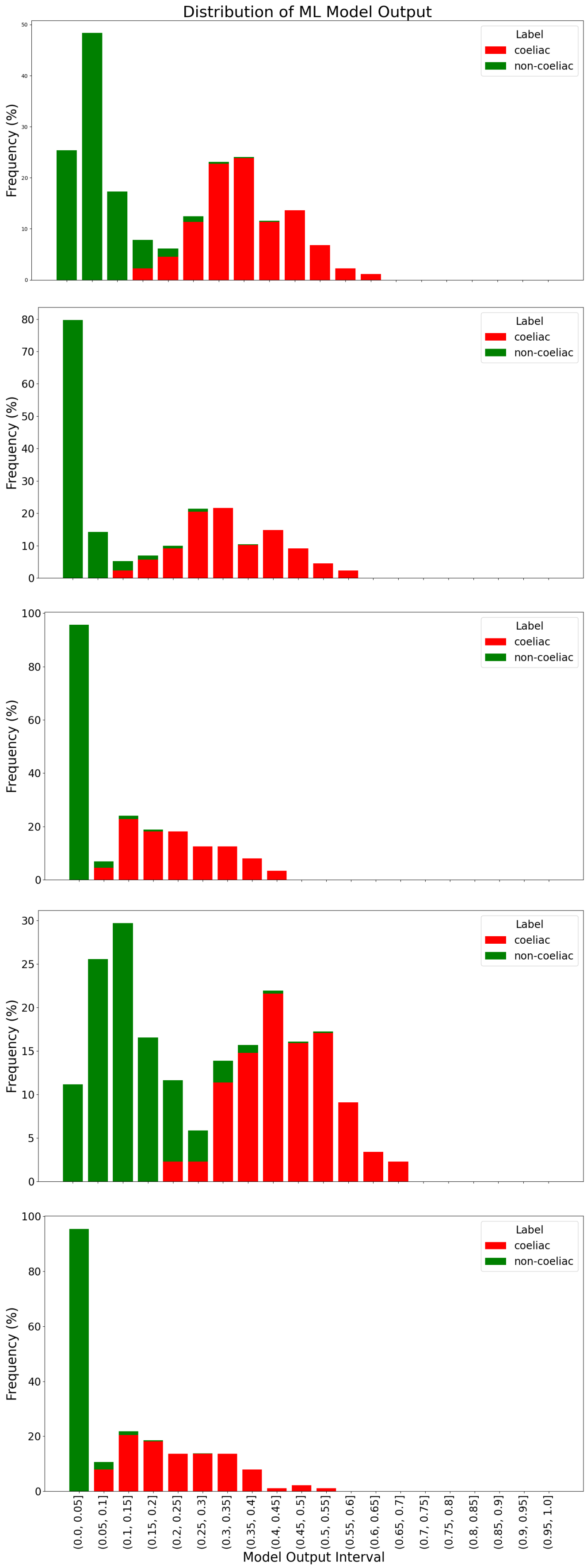

FIG. S2. Distribution of the model output over all images on the test sets for the five cross validation models, separated by existing clinical diagnosis.

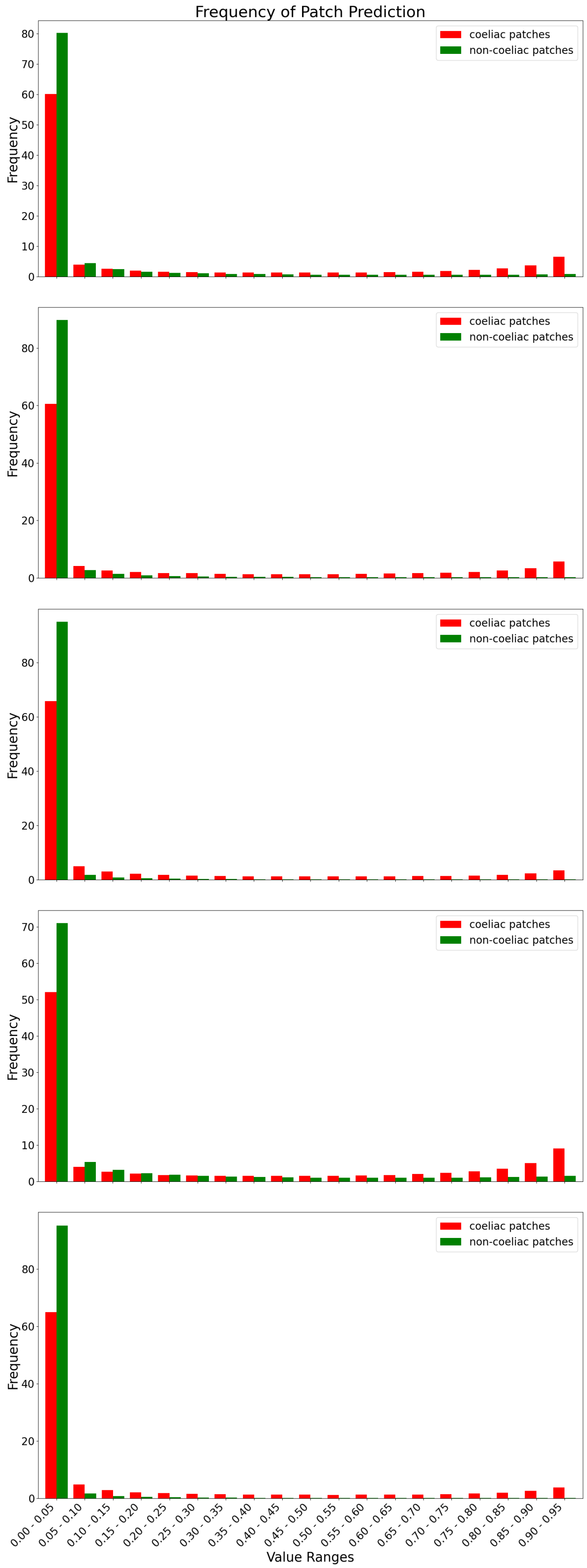

FIG. S3. Distribution of the model output over all patches on the test sets for the five cross validation models, separated by existing clinical diagnosis.

Appendix H: Explainability

We now demonstrate in Figures S4 - S7 how the five cross-fold validation models vary in their predictions by including heatmaps of all five models on the same images used in the main paper. In the main paper we ordered all coeliac cases from the test set based on how one cross-validation model (picked at random) classified the cases. We picked the 20th, 40th, 60th, and 80th percentile case in terms of confidence in a coeliac disease diagnosis. The outermost patches are often classified as normal; we hypothesis that this is caused by them containing very little tissue and a lot of background.

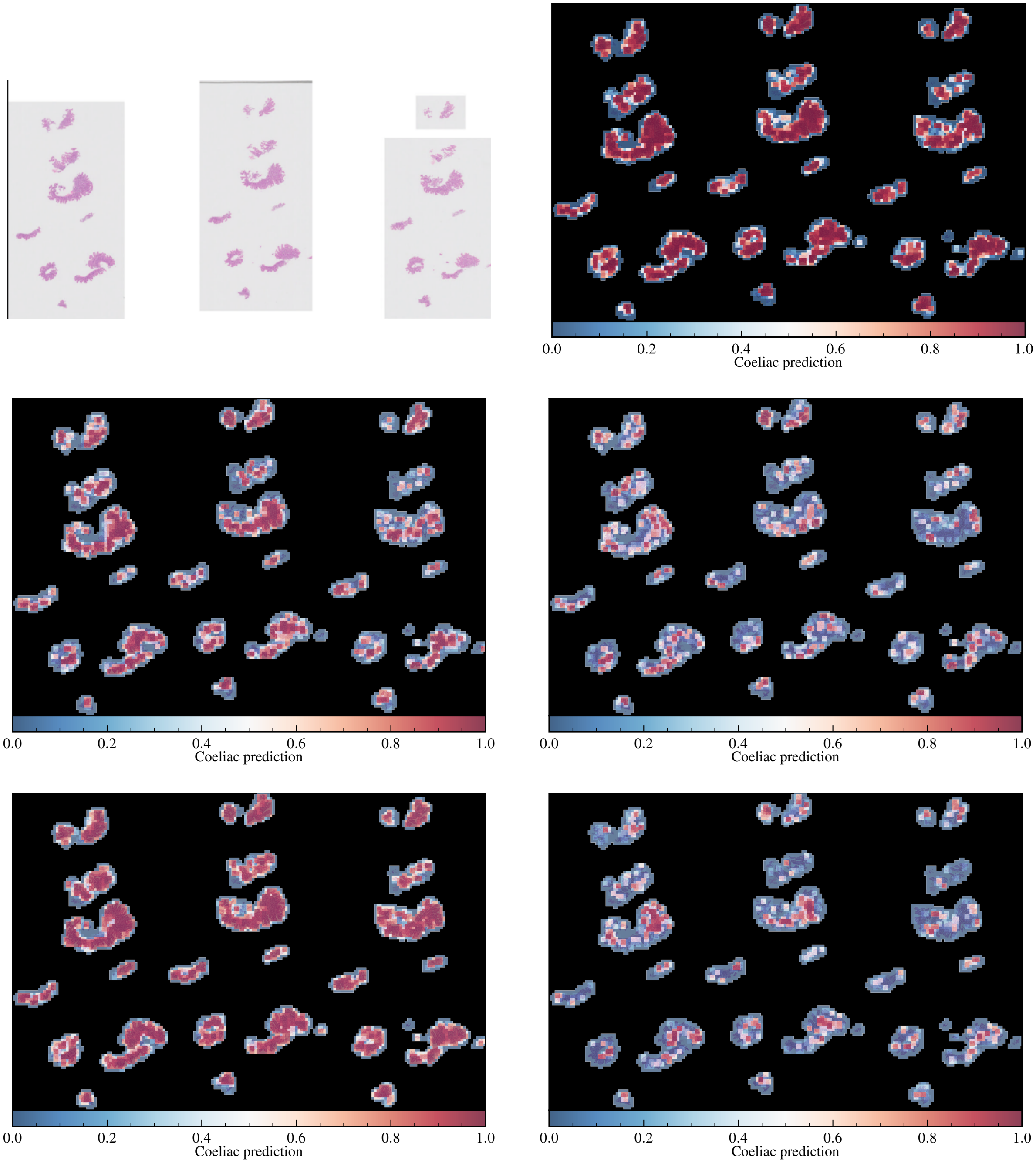

FIG. S4. Heatmaps for the five cross-validation models on a coeliac case.

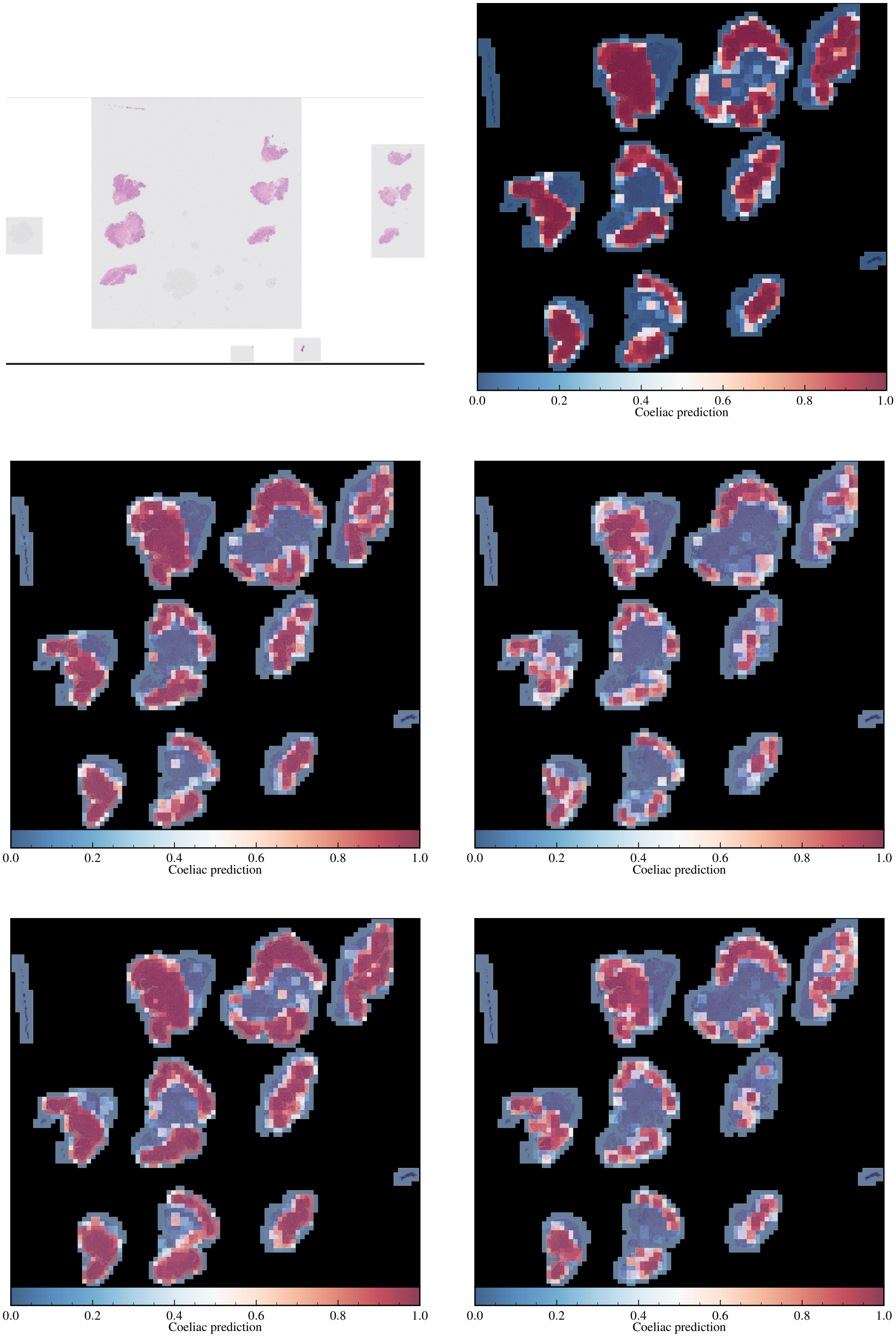

FIG. S5. Heatmaps for the five cross-validation models on a coeliac case.

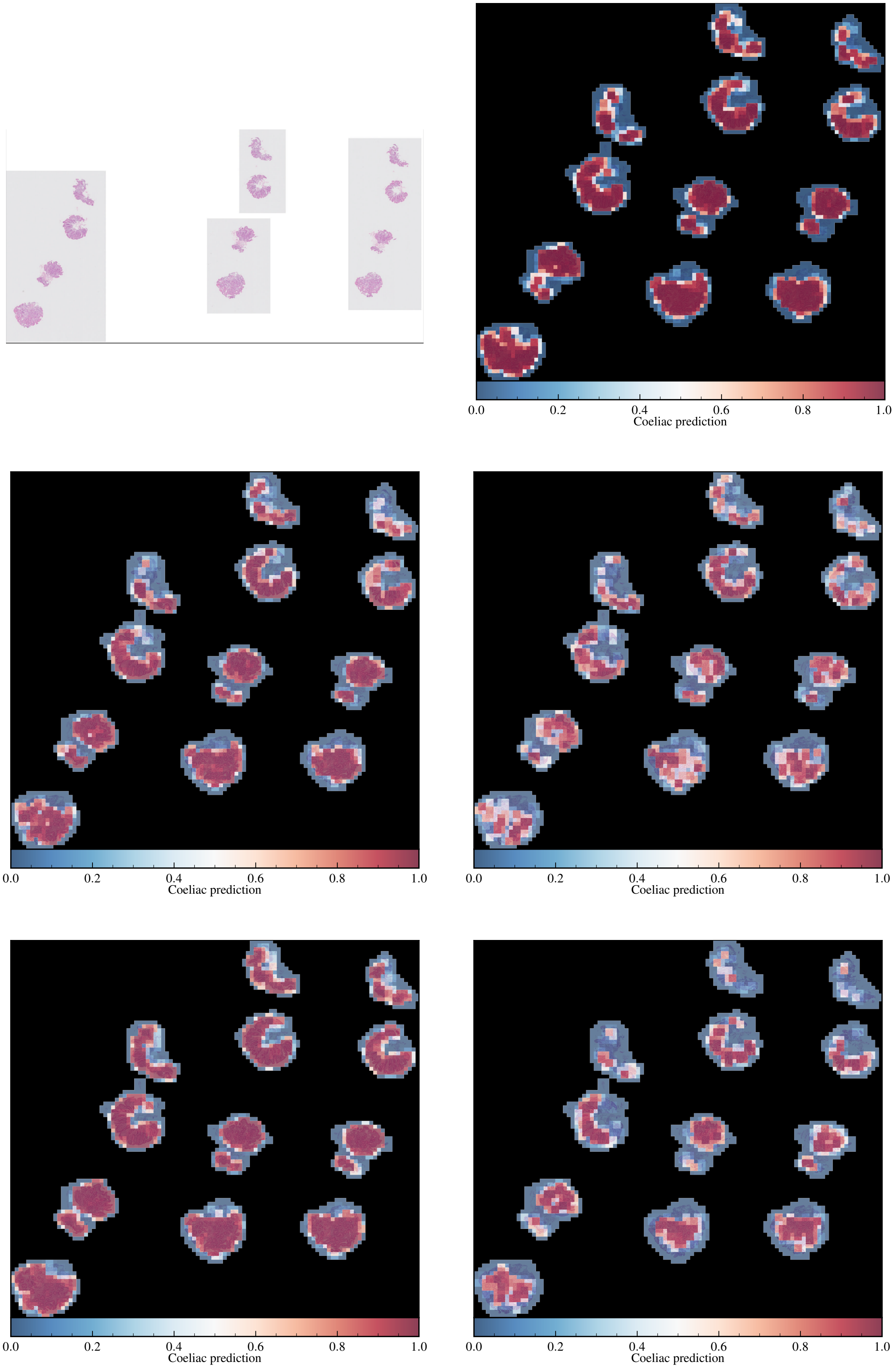

FIG. S6. Heatmaps for the five cross-validation models on a coeliac case.

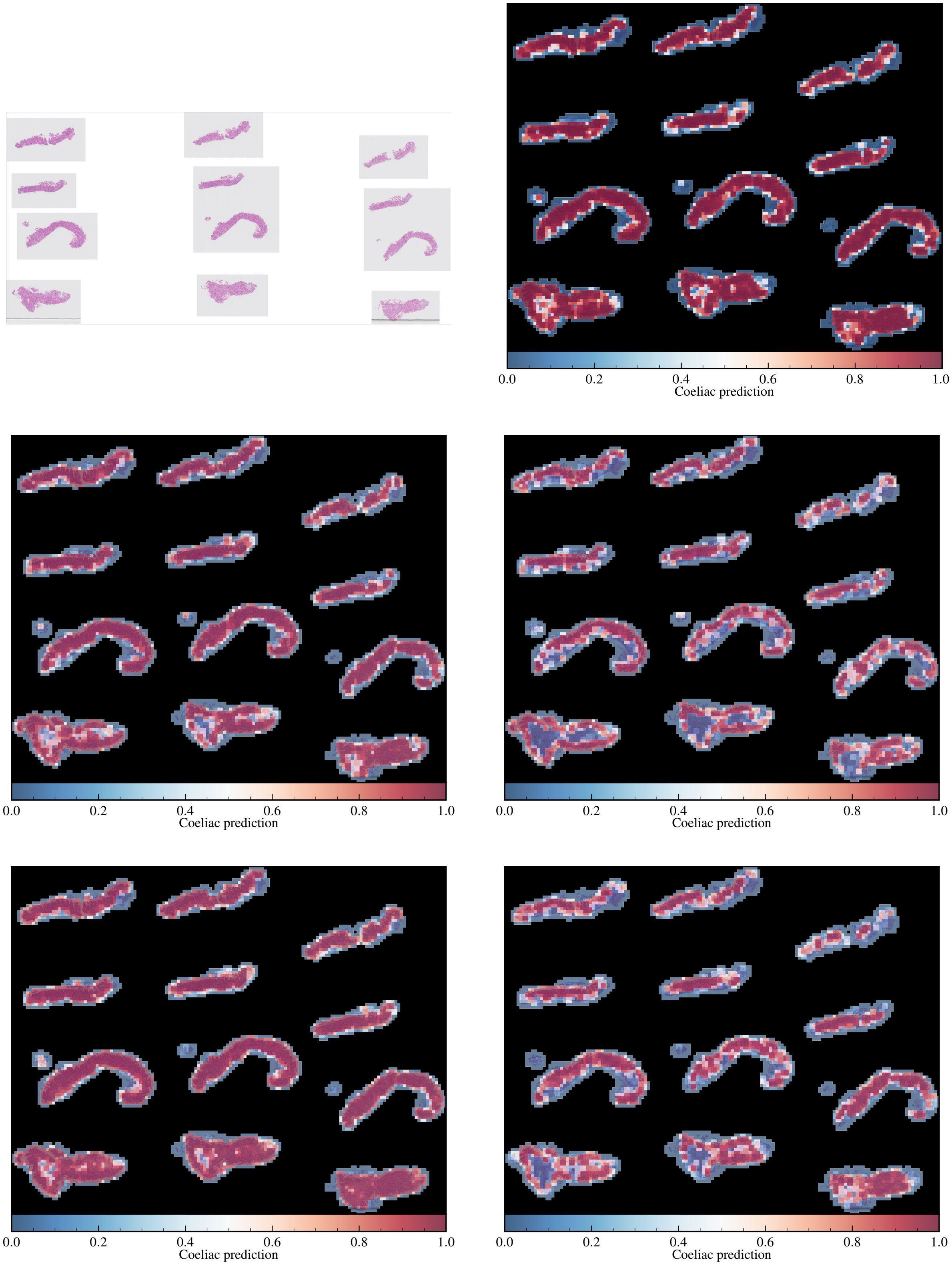

FIG. S7. Heatmaps for the five cross-validation models on a coeliac case.

Appendix I: Patches

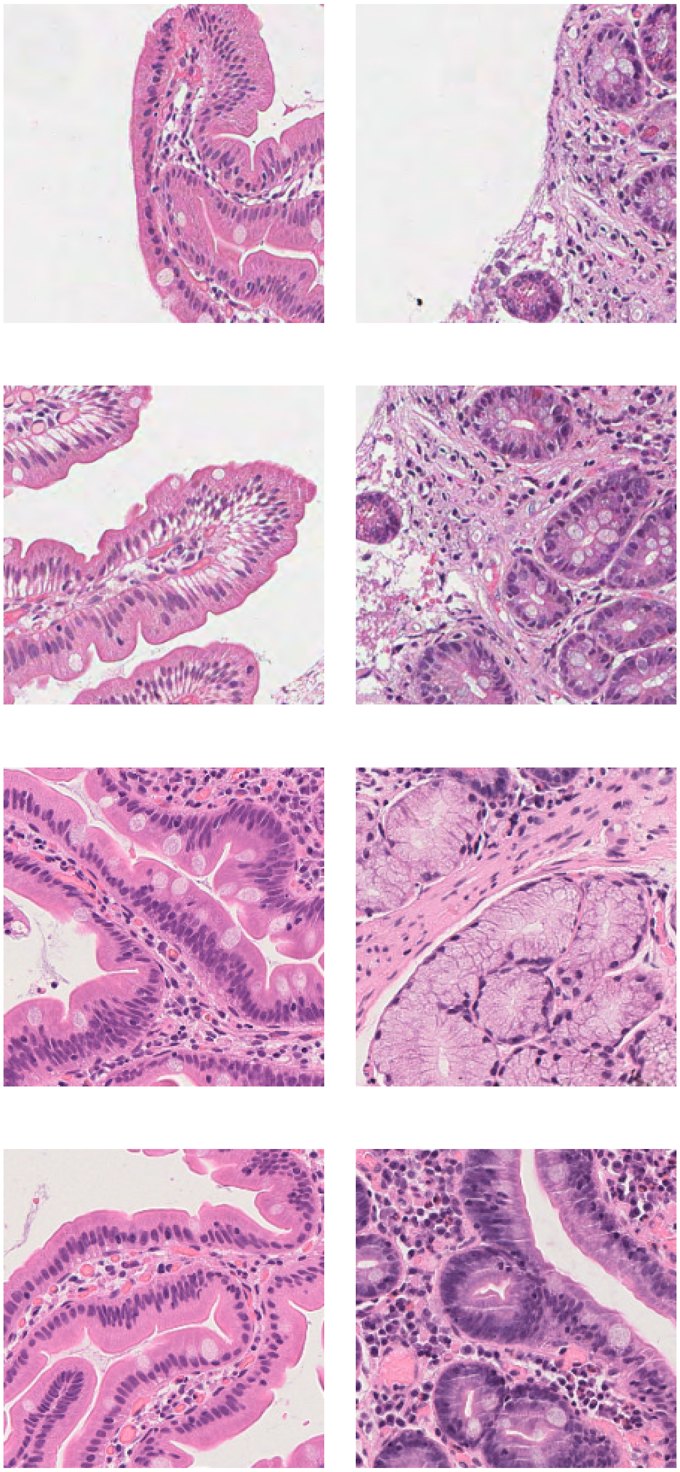

FIG. S8. Eight example patches at 10x magnification. Patches in the left column come from coeliac cases and patches on the right from normal cases. The top two rows contain patches from Addenbrookes cases, and the bottom two, contain patches from North Tees WSIs. We chose 10x magnification, as one can clearly see individual cells, as well as the more macro structures, such as villi and crypts.

[1] P. Zhou, J. Feng, C. Ma, C. Xiong, S. C. H. Hoi, *et al.*, Towards theoretically understanding why sgd generalizes better than adam in deep learning, Advances in Neural Information Processing Systems **33**, 21285 (2020).

[2] B. Schreiber, J. Denholm, J. Gilbey, C.-B. Schönlieb, and E. Soilleux, Stain normalization gives greater generalizability than stain jittering in neural network training for the classification of coeliac disease in duodenal biopsy whole slide images, Journal of Pathology Informatics **14**, 100324 (2023).

[3] F. Ciompi, O. Geessink, B. E. Bejnordi, G. S. De Souza, A. Baidoshvili, G. Litjens, B. Van Ginneken, I. Nagtegaal, and J. Van Der Laak, The importance of stain normalization in colorectal tissue classification with convolutional networks, in *2017 IEEE 14th International Symposium on Biomedical Imaging (ISBI 2017)* (IEEE, 2017) pp. 160–163.

[4] M. Salvi, F. Molinari, U. R. Acharya, L. Molinaro, and K. M. Meiburger, Impact of stain normalization and patch selection on the performance of convolutional neural networks in histological breast and prostate cancer classification, Computer Methods and Programs in Biomedicine Update **1**, 100004 (2021).

[5] J. Denholm, B. Schreiber, S. Evans, O. Crook, A. Sharma, J. Watson, H. Bancroft, G. Langman, J. Gilbey, C.-B. Schönlieb, M. Arends, and E. Soilleux, Multiple-instance-learning-based detection of coeliac disease in histological whole-slide images, Journal of Pathology Informatics **13**, 100151 (2022).

[6] J. W. Wei, J. W. Wei, C. R. Jackson, B. Ren, A. A. Suriawinata, and S. Hassanpour, Automated detection of celiac disease on duodenal biopsy slides: A deep learning approach, Journal of Pathology Informatics **10**, 7 (2019).

[7] R. Sali, L. Ehsan, K. Kowsari, M. Khan, C. A. Moskaluk, S. Syed, and D. E. Brown, Celiacnet: Celiac disease severity diagnosis on duodenal histopathological images using deep residual networks, Proceedings - 2019 IEEE International Conference on Bioinformatics and Biomedicine, BIBM 2019 , 962 (2019).

[8] A. M. Gruver, H. Lu, X. Zhao, A. D. Fulford, M. D. Soper, D. Ballard, J. C. Hanson, A. E. Schade, E. D. Hsi, K. Gottlieb, and K. M. Credille, Pathologist-trained machine learning classifiers developed to quantitate celiac disease features differentiate endoscopic biopsies according to modified marsh score and dietary intervention response, Diagnostic Pathology **18**, 122 (2023).
